# Effects of Daytime Food Intake on Memory Consolidation during Sleep or Sleep Deprivation

**DOI:** 10.1371/journal.pone.0040298

**Published:** 2012-06-29

**Authors:** Nina Herzog, Alexia Friedrich, Naoko Fujita, Steffen Gais, Kamila Jauch-Chara, Kerstin M. Oltmanns, Christian Benedict

**Affiliations:** 1 Department of Psychiatry and Psychotherapy, University of Luebeck, Luebeck, Germany; 2 General and Experimental Psychology, University of Munich (LMU), Munich, Germany; 3 Department of Neuroscience, Uppsala University, Uppsala, Sweden; Hosptial Infantil Universitario Niño Jesús, Spain

## Abstract

Sleep enhances memory consolidation. Bearing in mind that food intake produces many metabolic signals that can influence memory processing in humans (e.g., insulin), the present study addressed the question as to whether the enhancing effect of sleep on memory consolidation is affected by the amount of energy consumed during the preceding daytime. Compared to sleep, nocturnal wakefulness has been shown to impair memory consolidation in humans. Thus, a second question was to examine whether the impaired memory consolidation associated with sleep deprivation (SD) could be compensated by increased daytime energy consumption. To these aims, 14 healthy normal-weight men learned a finger tapping sequence (procedural memory) and a list of semantically associated word pairs (declarative memory). After the learning period, standardized meals were administered, equaling either ∼50% or ∼150% of the estimated daily energy expenditure. In the morning, after sleep or wakefulness, memory consolidation was tested. Plasma glucose was measured both before learning and retrieval. Polysomnographic sleep recordings were performed by electroencephalography (EEG). Independent of energy intake, subjects recalled significantly more word pairs after sleep than they did after SD. When subjects stayed awake and received an energy oversupply, the number of correctly recalled finger sequences was equal to those seen after sleep. Plasma glucose did not differ among conditions, and sleep time in the sleep conditions was not influenced by the energy intake interventions. These data indicate that the daytime energy intake level affects neither sleep’s capacity to boost the consolidation of declarative and procedural memories, nor sleep’s quality. However, high energy intake was followed by an improved procedural but not declarative memory consolidation under conditions of SD. This suggests that the formation of procedural memory is not only triggered by sleep but is also sensitive to the fluctuations in the energy state of the body.

## Introduction

Previous studies have shown that human sleep enhances the conversion of labile, recently acquired information into stable, long-term memories, which means that volunteers showed greater savings for memory when sleeping versus nocturnal wakefulness after the learning process [1]–[5]. Such consolidation effects of sleep have been found for declarative and for procedural types of memory tasks. Declarative memories are accessible to conscious recollection (e.g., vocabulary) whereas procedural memories comprise skills and habits that are usually not available for conscious recollection (e.g., playing piano). Compared to sleep, staying awake produces an increased energy demand in that 24-h energy expenditure exceeds that of a regular 24-h sleep/wake cycle by ∼7% [6]. It is assumed that the difference in the 24-h energy expenditure is caused by the sleep stage slow-wave sleep (SWS, often referred to as deep sleep) during which the central-nervous system energy demand is significantly lower than during wakefulness [7]; [8]. In this view, supplying the organism with more energy than required during daytime may counteract the impaired memory consolidation that is typically seen under conditions of nocturnal wakefulness [3].

There is also evidence that both caloric consumption and food intake-related signals (e.g. insulin level in cerebrospinal fluid) affect memory processing in humans during wakefulness [9]–[12]. For instance, glucose administration has been shown to enhance memory performance in healthy young people, and this effect was observed at least up to 24 h after glucose administration [13]. Moreover, it has been shown that performance during memory tasks was associated with a substantial decrease of glucose concentrations in the extracellular fluid in rats [14]. A preliminary study has also demonstrated that hypoglycemia during sleep is accompanied by impairments of cognitive performance in type 1 diabetic and healthy humans [15]. However, there are no studies that have systematically examined whether the sleep-dependent consolidation of declarative and procedural memories is sensitive to variations in preceding daytime energy intake. Further, the question of whether the impairing effect of sleep loss on memory consolidation can be reversed by oversupplying energy has yet to be addressed.

Against this background, in the present study memory consolidation was tested in healthy male subjects participating in four conditions. Twice, they slept according to their habitual sleep-wake-cycle, twice they were sleep-deprived for one night. In each sleep condition [i.e. sleep and total sleep deprivation (TDS), respectively] they once received an undersupply of energy, and once an oversupply of energy on the preceding day.

## Methods

### Ethics Statement

The study protocol conformed to the Declaration of Helsinki and was approved by the ethics committee of the University of Lübeck. All participants gave written informed consent.

### Participants

Fourteen healthy non-smoking male volunteers [age (mean ± SEM): 23.3±0.5 years; body mass index: high energy intake/TSD: 23.01±0.47 kg/m^2^, low energy intake/TSD: 22.96±0.49 kg/m^2^, high energy intake/sleep: 22.97±0.47 kg/m^2^, low energy intake/sleep: 22.89±0.48 kg/m^2^; *F* (1,13)  = 0.06; *p*  = 0.82 for high/low energy intake * sleep/wake ANOVA interaction] were included in the analyses. Overall, 16 subjects participated in the study but due to technical problems during the memory tasks, two of them were excluded from analyses. All subjects had a regular self-reported sleep-wake rhythm during the 6 weeks before the experiments and were not on any medication. Acute illness was excluded by physical examination and routine laboratory testing (e.g., fasting plasma glucose, serum triglycerides, serum urea, serum creatinin, serum gamma-glutamyl-transferase, white blood cell count). During the week before each experiment, participants were instructed to go to bed between 2300 h and 2330 h, to get up by 0700 h in the morning, and not to take any naps during the day. General sleep disturbances were excluded by monitoring sleep patterns in a separate adaptation night that also served to habituate subjects to the experimental laboratory environment.

### Experimental Design

According to a randomized, balanced cross-over design, each subject participated in four experimental conditions: high vs. low energy intake followed by regular sleep and high vs. low energy intake followed by TSD. To prevent possible anticipatory effects, subjects did not receive information regarding whether their session would involve sleep or wakefulness until 2100 h in the evening on the day of testing.

After an overnight fast, subjects arrived in the sleep laboratory at 0715 h and body weight was measured. Body weight did not differ among the conditions. Then, subjects participated in a memory-encoding session between 0800 h and 0830 h (see below). From 0900 h until 2300 h on the same day, subjects were either under- or oversupplied with calories, followed by either sleep or total sleep deprivation. During the day the participants were allowed to spend the time with non-arousing movies, games and books. In the sleep conditions, lights were turned off at 2300 h and subjects were awakened at ∼0600 h. During TSD, lights were on (∼300 lux) and subjects were kept awake and continuously monitored by the experimenters. The participants stayed in bed during the whole night, and were allowed to spend the night with non-arousing activities comparable to the daytime activity. Drinking and food intake were not allowed.

**Figure 1:**
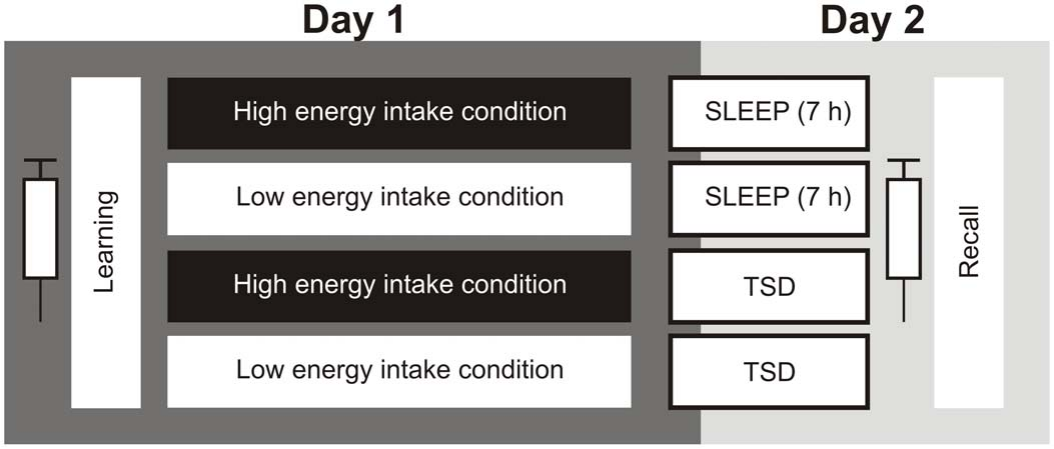
Experimental protocol. According to a randomized, balanced cross-over design, each subject participated in four experimental conditions: high caloric intake followed by regular sleep, low caloric intake followed by regular sleep, high caloric intake followed by total sleep deprivation (TSD), and low caloric intake followed by total sleep deprivation. After an overnight fast, in the morning of day 1, subjects had to learn both a declarative (word pair associates) and procedural memory task (finger sequence tapping) at 0800 h. Thereafter, they were provided with three meals (0900 h, 1300 h, 1900 h) and three additional drinks (1030 h, 1500 h; 1700 h), equaling ∼ 50% of their estimated individual total energy expenditure ( =  low energy intake conditions). In the high caloric intake conditions, the energy content of all meals and drinks provided were increased by means of maltodextrin (taste- and odorless carbohydrate), corresponding to ∼ 150% of the estimated individual total energy expenditure. In half of the conditions, in the subsequent night sleep was permitted from 2300 h (lights off) to 0600 h (lights on); in the other conditions, subjects remained awake throughout the whole experimental period. In the morning of day 2, i.e., after a post-treatment consolidation interval of 26 hours, recall of word pairs and procedural skill performance was tested. Syringe symbols denote blood samplings for the determination of plasma glucose.

In the morning after the sleep intervention, following a liquid breakfast meal at 0800 h (1256 kJ), subjects were asked to recall the memories they learned the day before (1030 h). A liquid breakfast meal before the recall test was given to compensate for possible energy deficit due to TSD. In order to avoid any strenuous physical stress during the entire experiments, subjects rested either in bed in a supine or a sitting position. All sessions were separated by at least two weeks. A schematic of the experimental design can be found in **Figure 1**.

### Energy Intake Intervention

Following memory encoding, participants received three regular standard meals and three additional drinks (detailed macronutrient composition can be found in **Table 1**). The total amount of meal intake (3777 kJ in sum) equalled ∼40% of the estimated daily energy expenditure. In order to produce the low energy intake condition (50% of the individual energy expenditure), three additional drinks were enriched with the carbohydrate maltodextrin (MaltoCal^19^, MetaX, Friedberg, Germany). Calculation of the individual energy expenditure was conducted according to the Harris-Benedict equation, and a physical activity factor of 1.2 was taken into account (corresponding to sedentary activities). In the high energy conditions, meals and drinks were enriched with maltodextrin to a calorie content of 13816 kJ on average (∼ 150% of the estimated total energy expenditure). Importantly, maltodextrin is a tasteless and odourless nutrient, thereby ensuring that the subjects were unaware of the respective energy intake intervention. Supporting the view that the participants were blind to the energy intake intervention, the addition of maltodextrin to the test meals did not affect satiety ratings, as assessed by visual analogue appetite ratings [high energy intake/TSD vs. low energy intake/TSD: 2.84±0.62 vs. 3.27±0.46, *p*  = 0.59; high energy intake/sleep vs. low energy intake/sleep: 2.77±0.41 vs. 2.80±0.45, *p*  = 0.94; *F* (2,24)  = 0.83; *p*  = 0.42 for high/low energy intake * sleep/wake * time ANOVA interaction].

**Table 1:** Macronutrient composition of test meals provided on the day before sleep or total sleep deprivation.

Time of day	Meal	Total energy, in kJ	F, in %kJ	CH, in % kJ	P, in %kJ
0900 h	Breakfast	1139	23	63	14
1030 h	Tea (400 ml)	0	0	0	0
1300 h	Lunch	1876	19	54	27
1500 h	Tea (400 ml)	0	0	0	0
1700 h	Tea (400 ml)	0	0	0	0
1900 h	Dinner	762	17	61	22

On the day before sleep or total sleep deprivation, participants received three regular standard meals and three additional drinks. The total amount of meal intake (3777 kJ in sum) equalled ∼ 40% of the estimated daily energy expenditure. In order to produce the low energy intake condition (50% of the individual energy expenditure), the three additional drinks were enriched with the taste- and odourless carbohydrate maltodextrin. In the high caloric conditions (150% of the individual energy expenditure), all main meals as well as the additional drinks were also enriched with maltodextrin, leading to a calorie content of 13816 kJ on average. All values were rounded. Abbreviations: CH, carbohydrate; F, fat; P, protein.

### Sleep Monitoring

Sleep stages were monitored offline with polysomnographic recordings, following standard criteria [16]. Polysomnographic recordings were performed by using a digital EEG device connected to surface electrodes. The electrodes were attached to the scalp (electroencephalogram, EEG), around the eyes (horizontal and vertical electrooculogram, EOG), and to the chin (electromyogram, EMG) for standard measurements. For each night, sleep onset (with reference to lights off at 2300 h), total sleep time, duration of the different sleep stages (wake; stages 1, 2, 3, and 4; and REM sleep), and latency of sleep stages with reference to sleep onset were determined. Slow-wave sleep was defined as the time spent in non-REM-sleep stages 3 and 4.

### Memory Tests

To investigate declarative and procedural memory consolidation, a word-pair associate learning task and a finger sequence tapping task were administered to the subjects. These specific memory tests were chosen because performance on them has repeatedly been shown to benefit from sleep [5]; [17]; [18]. Upon arrival at 0715 h on the first day, subjects learning session started at 0800 h, i.e., before the energy intake intervention period was started. Retrieval testing took place at 1030 h the next morning.

#### Word-pair associates [17]; [18]

Participants were instructed to learn 40 semantically related word pairs of German nouns standardized with respect to word frequency, length, emotionality, meaningfulness, and concreteness (e.g. car & street). Word pairs were presented visually for 5s each. After presentation of all word pairs, subjects were asked to recall orally the second word in a pair upon presentation of the first word (immediate cued recall). During learning, the subject’s response was always followed by a presentation of the correct response word for 1 sec. The list was presented repeatedly in a different order until the subject had correctly recalled at least 24 words (60% criterion). During retrieval testing after either sleep or sleep deprivation, subjects were again asked to recall the word pairs using the same cued recall procedure that had been applied during the learning phase, but this time without any feedback. Retention performance was defined by the percentage of recalled word pairs during retrieval, with performance on the learning trial set to 100%. Subjects were tested using parallel tests, which were given in a randomly sequenced order.

#### Finger sequence tapping task [5]; [17]; [19]

The finger sequence tapping task requires the subjects to press repeatedly a five element sequence (e.g. 3-4-1-2-4) on a keyboard, using the fingers of the non-dominant hand, as fast and as accurately as possible. The numeric sequence was displayed on the screen at all times to keep working memory demands at a minimum. Each key press resulted in a white dot in the center of the screen. At learning, subjects performed twelve 30 s trials, each interrupted by 30 s breaks (overall task duration: 12 min). Retrieval testing included three trials. For each 30 s trial the number of correctly tapped sequences was determined. Retention performance was determined by the average number of correctly tapped sequences across the three trials at retrieval, with the average performance on the last three trials at learning set to 100%. Subjects were tested using parallel five element sequences which were given in a random order.

**Table 2:** Plasma glucose concentrations and memory performance.

	SleepHigh energyintake	SleepLow energyintake	TSDHigh energyintake	TSDLow energyintake	ANOVASleep/TSD	ANOVAHigh/Low energy intake	ANOVASleep/TSD * Energy intake
**Plasma glucose (mmol/l)**							
*Before Learning*	5.0±0.2	5.1±0.1	4.8±0.1	5.0±0.1	*F* (1,13) = 1.01, *p* = 0.33	*F* (1,13) = 1.13, *p* = 0.31	*F* (1,13) = 0.92, *p* = 0.35
*Before Recall*	5.4±0.3	5.7±0.2	5.6±0.2	6.0±0.4	*F* (1,13) = 0.49, *p* = 0.50	*F* (1,13) = 1.63, *p* = 0.22	*F* (1,13) = 0.04, *p* = 0.84
**No. of correct word pairs**							
*Learning*	27.9±0.7	27.9±0.8	30.0±0.9	28.1±1.1	*F* (1,13 ) = 1.81, *p* = 0.20	*F* (1,13) = 1.04, *p* = 0.33	*F* (1,13) = 1.33, *p* = 0.27
*Trials to criterion*	1.7±0.2	1.8±0.2	1.8±0.2	1.4±0.1	*F* (1,13) = 1.37, *p* = 0.26	*F* (1,13) = 0.78, *p* = 0.39	*F* (1,13) = 2.49, *p* = 0.14
*Recall*	25.4±1.0	25.7±1.3	25.4±1.5	24.0±1.7	*F* (1,13) = 0.68, *p* = 0.43	*F* (1,13) = 0.24, *p* = 0.63	*F* (1,13) = 0.59, *p* = 0.46
*Recall/Learning (in %)*	91.4±3.3	92.3±4.2	84.2±3.2	85.2±4.7	*F* (1,13) = 4.94, *p* = **0.05**	*F* (1,13) = 0.10, *p* = 0.76	*F* (1,13) = 0.00, *p* = 0.99
**No. of correct sequences**							
*Learning*	19.9±1.6	19.7±1.0	20.1±1.3	19.7±1.7	*F* (1,13) = 0.01, *p* = 0.91	*F* (1,13) = 0.12, *p* = 0.73	*F* (1,13) = 0.01, *p* = 0.93
*Recall*	22.4±1.7	22.9±1.2	23.4±1.6	20.3±1.9	*F* (1,13) = 0.57, *p* = 0.46	*F* (1,13) = 2.33, *p* = 0.15	*F* (1,13 ) = 2.47, *p* = 0.14
*Recall/Learning (in %)*	114.5±5.0	116.4±3.6	116.4±3.1	102.6±2.7	*F* (1,13) = 1.99, *p* = 0.18	*F* (1,13) = 6.93, *p* = **0.02**	*F* (1,13) = 4.55, *p* = **0.05**

Plasma glucose concentrations and memory performance on a word pair task and finger sequence tapping task before and after either one night of regular sleep (2300 h –0600 h) or total sleep deprivation (TSD). Before sleep or TSD but after learning (ie., 0800 h, Day 1), all subjects were provided with three meals (0900 h, 1300 h, and 1900 h) and three additional drinks (1030 h, 1500 h, and 1700 h), corresponding to 50% of their estimated individual total energy expenditure (‘low energy intake conditions’). In the high caloric intake conditions, the energy content of all meals and drinks provided were increased by means of maltodextrin (taste- and odorless carbohydrate), corresponding to 150% of the estimated individual total energy expenditure. Delayed recall was tested after consumption of a defined amount of calories at 1030 h in the morning on the day after sleep or TSD. Data are means ± SEM; significant repeated ANOVA measures effects are indicated in bold (i.e.; *p*≤0.05).

### Plasma Glucose Measurements

To control for plasma glucose concentrations, blood was drawn via an intravenous forearm catheter immediately before learning (i.e., 0800 h on the first day) and retrieval (i.e., 1030 h on the day after sleep or sleep deprivation). A clinical routine assay was used for the determination of plasma glucose (Abbott, Abbott Park, IL).

### Statistical Analyses

For statistical evaluation, SPSS version 20.0 (SPSS Inc, Chicago, IL) was used. Data are presented as means ± SEMs. Statistical analyses were based on analyses of variance (ANOVA) including the repeated-measures factors Sleep/Total sleep deprivation (TSD) and Low/High energy intake. ANOVAs were followed by post hoc comparisons using Student’s t tests for paired data. Overall, a two-sided *p*≤0.05 was considered significant. Normal distribution of the data was assured by using the Kolmogorov-Smirnov test (*p*>0.750 for all comparisons).

**Figure 2:**
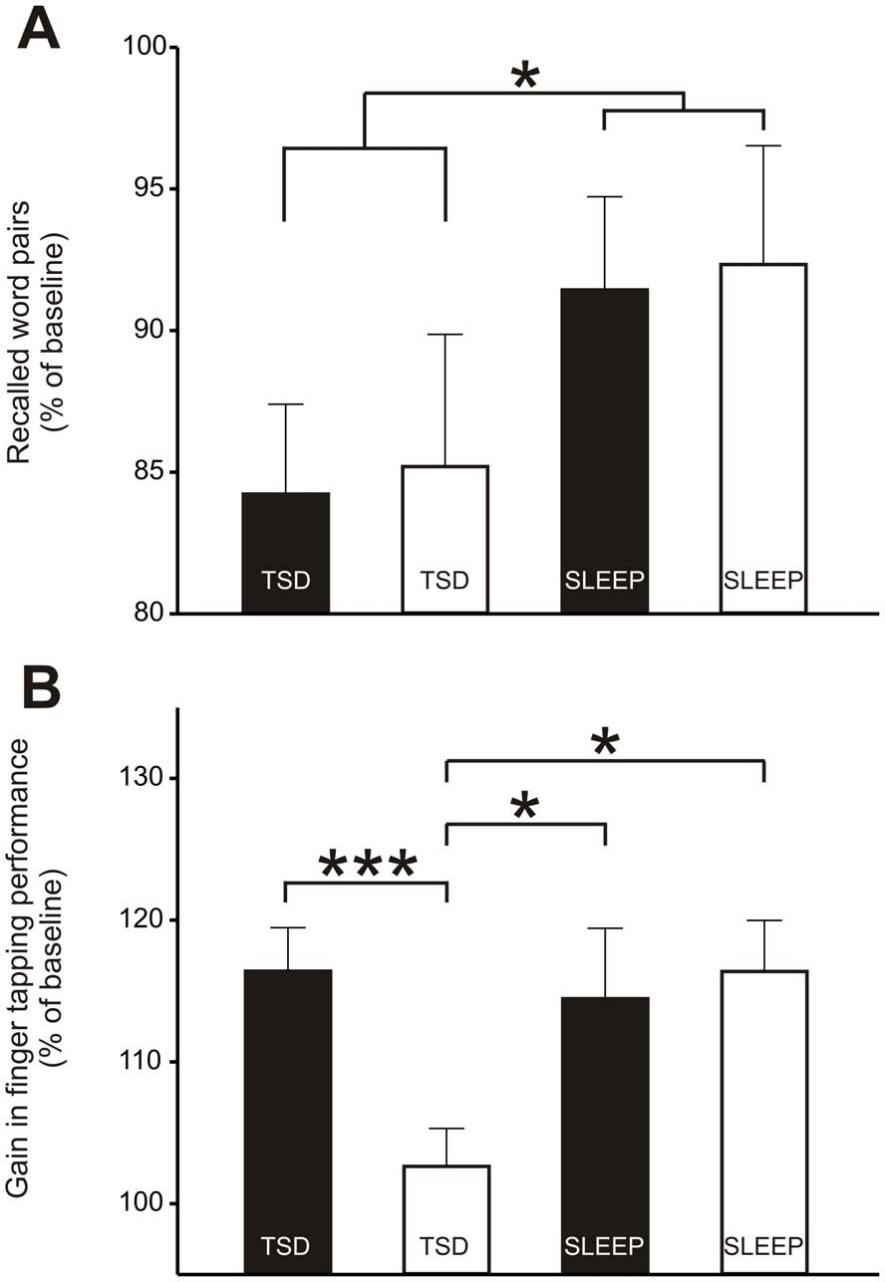
The influence of energy intake on procedural and declarative memory consolidation during sleep and total sleep deprivation. Values are presented as means ± SEMs. **(A)** Number of correctly recalled word pairs in the morning after one night of either total sleep deprivation (TSD) or 7-h sleep opportunity. On the day before sleep or nocturnal wakefulness but after learning (0800 h), subjects (n = 14) were either over- (black bar) or undersupplied (white bar) with calories, ie., they consumed either ∼50% or ∼150% of their estimated individual total energy expenditure. Retention performance is indicated by the percentage of word pairs recalled at retrieval (1030 h in the morning after sleep or TSD), with performance on the criterion trial during learning set to 100%. Repeated-measures ANOVA revealed a significant main effect for Sleep/TSD (*p*  = 0.05). **(B)** Overnight gain in finger tapping skill is indicated by the average percentage of the number of correct sequences per 30 s trial on the three trials at retrieval testing (1030 h in the morning after sleep or TSD), with the average performance at the end of learning (trial 10–12) set to 100%. Repeated-measures ANOVA produced a significant interaction between the Sleep/TSD conditions and the energy intake intervention (*p*  = 0.05). *p≤0.05, ***p<0.001 for pairwise comparisons between conditions.

## Results

### Memory Consolidation

The results of the repeated-measures ANOVA are given in table 2. Learning performance on the declarative memory task, i.e., word pair learning, was comparable between the sleep and TSD conditions in terms of the number of correct word pairs recalled during the criterion trial (**Table 2**). Likewise, the number of trials needed to reach the criterion of 24 correctly remembered word pair associates (i.e., 60%) did not differ between the conditions (**Table 2**). However, independent of the energy intake preceding sleep or TSD subjects were able to remember more word pairs the next morning when they slept after learning than they did after one night of TSD (**Table 2** and **Figure 2**).

During learning, performance on the finger tapping task was not different between the sleep and TSD conditions (**Table 2**). Comparisons of finger tapping at retrieval (expressed as percentage of the learning performance) produced a significant interaction between the Sleep/TSD conditions and the energy intake intervention, whereas no significant Sleep/TSD main effect was found (**Table 2**). Post hoc comparisons revealed that high energy intake preceding TSD was followed by a gain in finger tapping performance which was comparable to that in the sleep conditions (*p*≥0.75 for all pair-wise comparison; **Figure 2** **and** **Table 2**). In contrast, low energy intake before the SD condition led to lower finger tapping performance compared to all other conditions (*p*<0.05 for all pair-wise comparison; **Figure 2** **and** **Table 2**). Moreover, the gain in finger tapping performance seen between learning and retrieval in the sleep conditions was unaffected by energy intake (*p*≥0.73 for all pair-wise comparison; **Figure 2** **and** **Table 2**).

### Plasma Glucose Concentrations and Sleep Recordings

Plasma glucose values before learning (i.e., after arrival on the first day) and retrieval did not differ between conditions (**Table 2**). Sleep was typical for laboratory conditions, and was not influenced by the energy intake in the preceding wake period (**Table 3**).

**Table 3:** Sleep data after either low or high energy intake in the preceding wake period.

	High energy intake	Low energy intake	*P* -Value
Total sleep time	397.4±5.5	396.1±4.8	0.89
Sleep onset latency	25.6±5.7	36.0±8.9	0.19
SWS onset latency	19.5±1.9	24.5±5.8	0.44
REM onset latency	98.2±12.1	114.2±22.5	0.56
Wake	11.6±3.1	7.8±2.5	0.28
Stage 1 sleep	21.6±3.8	19.2±4.8	0.46
Stage 2 sleep	213.3±10.5	218.7±7.2	0.65
Stage 3 sleep	36.6±3.4	41.0±5.6	0.35
Stage 4 sleep	40.6±6.5	34.8±5.0	0.43
REM	68.7±8.2	66.3±7.4	0.82
Movement time	3.3±1.7	8.4±7.4	0.52

Sleep data are shown as means ± SEM, and were recorded in the night following either an over- or undersupply with calories, i.e., subjects (n = 14) consumed either 150% or 50% of their estimated individual total energy expenditure. Total sleep time, time spent awake, stage 1 sleep, stage 2 sleep, stage 3 sleep, stage 4 sleep, rapid eye movement (REM) sleep, and movement arousal are given in minutes, and latency of the first period of slow-wave-sleep (SWS) and REM sleep (with reference to sleep onset) are shown. Pairwise comparisons were specified by Student t-tests.

## Discussion

In the present study we show, as indicated by the higher number of correctly recalled word pairs in the sleep conditions, that the consolidation of declarative memory is better after sleep than after total sleep deprivation (TSD). This effect was not sensitive to variations in energy intake on the day preceding sleep. In contrast, energy oversupply preceding TSD induced a procedural memory gain which was comparable to that in the sleep conditions. Such an effect was not seen under conditions of energy undersupply. These data suggest on the one hand that memory consolidation during sleep is robust against acute fluctuations in energy states of the body, such as dieting (i.e. restricting calorie consumption) or overeating. On the other hand, one could speculate that production of procedural long-term memory occurs not only after sleep but also under conditions of wakefulness, as long as enough energy is supplied.

Declarative memory savings (i.e., the number of word pairs recalled correctly after the post-learning retention interval) were greater after sleep than after wakefulness, which is in line with the existing literature [3]; [18]; [20]; [21]. However, the beneficial effect of sleep on declarative memory consolidation was not influenced by the energy intake on the day preceding sleep. Moreover, declarative memory savings were not preserved when sleep-deprived subjects had been oversupplied with energy. This pattern suggests that the formation of long-term memories for declarative information appears to be due to sleep processes that are not energy-dependent. In line with this assumption, studies have shown that even very short sleep periods (e.g., ∼40 min) after learning improve declarative memory consolidation as compared to wakefulness [1].

In contrast to that of declarative memory, the consolidation of procedural skills (i.e., the number of finger sequences recalled correctly after the post-learning retention interval) was influenced by daytime energy intake. Providing more energy than needed on the day before nocturnal sleep deprivation was followed by a preservation of procedural skills the next morning that resembled that found after sleep. One possible explanation might be that the impairing effect of low energy intake on procedural memory consolidation compared to high energy intake under conditions of TSD, is due to the energy deficit produced by staying awake [6], thus leaving not enough energy for consolidation. In that sense, the enhancing role of sleep in strengthening procedural memory might be a by-product of sleep’s low energy demand as compared to wakefulness [7]; [8]. Supporting this assumption, previous studies have shown that improvements of finger sequence tapping performance are more robust after one night of sleep, as compared with that seen after short naps [21]. Bearing this pattern in mind, the gain in finger sequence performance that is typically observed after post-learning sleep may be therefore secondary to the energy saving effects of sleep. An alternative interpretation of our finding could be that sleep deprivation leads to fatigue and thus impairs performance during memory recall. A higher level of available energy could then be beneficial by improving procedural task performance. However, due to the fact that our subjects had consumed a defined amount of calories at breakfast before memory retrieval was tested in all four conditions, this assumption appears less likely.

### Limitation

The main limitation of our study is that we were not able to show a gain in procedural memory, independent of energy intake, as seen in other studies [4]. It is conceivable that the sample size was too small to find a significant sleep/TSD main effect. Additionally, the procedural and declarative memory tests used should be examined in future studies to replicate our results. For practical reasons, women were not included, as this would have required additional control of menstrual hormones which are well-known to modulate both sleep and energy metabolism. Thus, future studies should explore how the menstrual cycle influences sleep-dependent memory consolidation, and whether this depends on the energy intake on the day preceding sleep.

### Conclusion

Our data suggest that procedural memory can also be sufficiently consolidated online, during a wake retention period, as long as enough energy is available during the retention interval. Further, our study demonstrates that calorie intake before sleep does not affect subsequent sleep-dependent consolidation of declarative memory. Additionally, in line with previous findings [22], changes in daytime energy intake did not affect sleep architecture as revealed by the EEG recordings.
